# A meta-analysis of perventricular device closure of doubly committed subarterial ventricular septal defects

**DOI:** 10.1186/s13019-020-1062-0

**Published:** 2020-01-28

**Authors:** Jiang-Shan Huang, Kai-Peng Sun, Shu-Ting Huang, Qiang Chen, Liang-Wan Chen, Yur-Ren Kuo

**Affiliations:** 1 Department of Cardiovascular Surgery, Union Hospital, Fujian Medical University, Fuzhou, 350001 People’s Republic of China; 2 Department of Cardiac Surgery, Fujian Provincial Maternity and Children’s Hospital, affiliated hospital of Fujian Medical University, Fuzhou, 350001 People’s Republic of China; 3 Division of Plastic Surgery, Department of Surgery, Kaohsiung Medical University Hospital, 100 TzYou 1st Rd, Kaohsiung City, 80756 Taiwan

**Keywords:** Perventricular device closure, Doubly committed subarterial ventricular septal defect, Meta-analysis

## Abstract

**Background:**

To investigate the safety and efficacy of perventricular device closure of doubly committed subarterial ventricular septal defects (dcsVSDs).

**Methods:**

PubMed and Scopus were searched for studies in English that focused on perventricular device closure of dcsVSDs and were published up to the end of September 2019. We used a random-effects model to obtain pooled estimates of the success and complication rates.

**Results:**

A total of 9 publications including 459 patients with dcsVSDs were included. The median follow-up duration ranged from 2 months to 5 years, with the mean age of patients ranging from 6.1 months to 4.5 years. The pooled estimate of the overall success rate of device closure in the 9 studies was 0.89 (95% CI: 0.86–0.93, I^2^ = 26.5%, *P* = 0.208). Further meta-regression analysis indicated no significant correlation between the success rate and the following factors: publication year, sample size, study type, mean age, mean weight, mean VSD size, and ratio of device size/weight. The pooled rate of postoperative aortic regurgitation was 0.045 (95% CI: 0.018–0.071, I^2^ = 50.96%, *P* = 0.000). The pooled rate of follow-up aortic regurgitation (AR) was 0.001 (95% CI, − 0.003-0.004, I^2^ = 63.00%, *P* = 0.009.) The pooled estimated rate of severe intraoperative complications was 0.106 (0.073–0.140, I^2^ = 70.7%, *P* = 0.208). Postoperative and follow-up complications were rare. No occurrence of a complete atrioventricular block was reported up to the last follow-up visit.

**Conclusions:**

Perventricular device closure may be an alternative to conventional surgical repair in selected patients with dcsVSDs. The success rate was stable regarding the publication year and sample size, suggesting a relatively short learning curve and the technique’s potential for application.

## Introduction

Ventricular septal defects (VSDs) account for 20% of all forms of congenital heart defects, and approximately 5–7% of VSD cases are doubly committed subarterial VSDs (dcsVSDs) in Asian populations, which have a low tendency for spontaneous closure and a high incidence of aortic valve prolapse [1–4]. Early intervention is recommended for patients with dcsVSDs. Surgical repair of dcsVSDs under cardiopulmonary bypass (CPB) is considered the gold standard treatment. However, this procedure cannot avoid the potential risk of CPB-related complications, the need for a blood transfusion, and the presence of a surgical incision scar or prolonged recovery [5, 6]. With the improvement of technology and development of devices, transfemoral device closure of dcsVSDs has also been applied in some cardiac centers [7, 8]. However, such an approach is still limited by technical difficulty, caused by the special location of dcsVSDs, which results in longer radiation times and lower success rates. In recent years, perventricular device closure of dcsVSDs has been developed as an alternative to conventional surgical repair in China [9–17]. No meta-analysis focusing on perventricular device closure of dcsVSDs has been reported. This study aimed to obtain pooled estimates of the success and morbidity rates after perventricular device closure of dcsVSDs, based on a meta-analysis of the current literature. The results might further guide research on the risk factors for complications to achieve better outcomes with fewer complications.

## Methods

### Literature search strategy

A search of the English literature, from the start date of each database up to the end of September 2019, was conducted by 2 independent researchers using PubMed (MEDLINE), EMBASE, and the Cochrane Central Register of Controlled Trials with the following search terms: VSD, sub-arterial, mini-invasive, transthoracic, intraoperative, perventricular, and device closure. From this search list, studies investigating the results of perventricular device closure of dcsVSDs were identified. Reference lists of the included articles were further examined to identify other relevant studies. Excluded studies and the reasons for their exclusion were listed and examined by a third researcher.

### Study selection and quality assessment

**Fig. 1:**
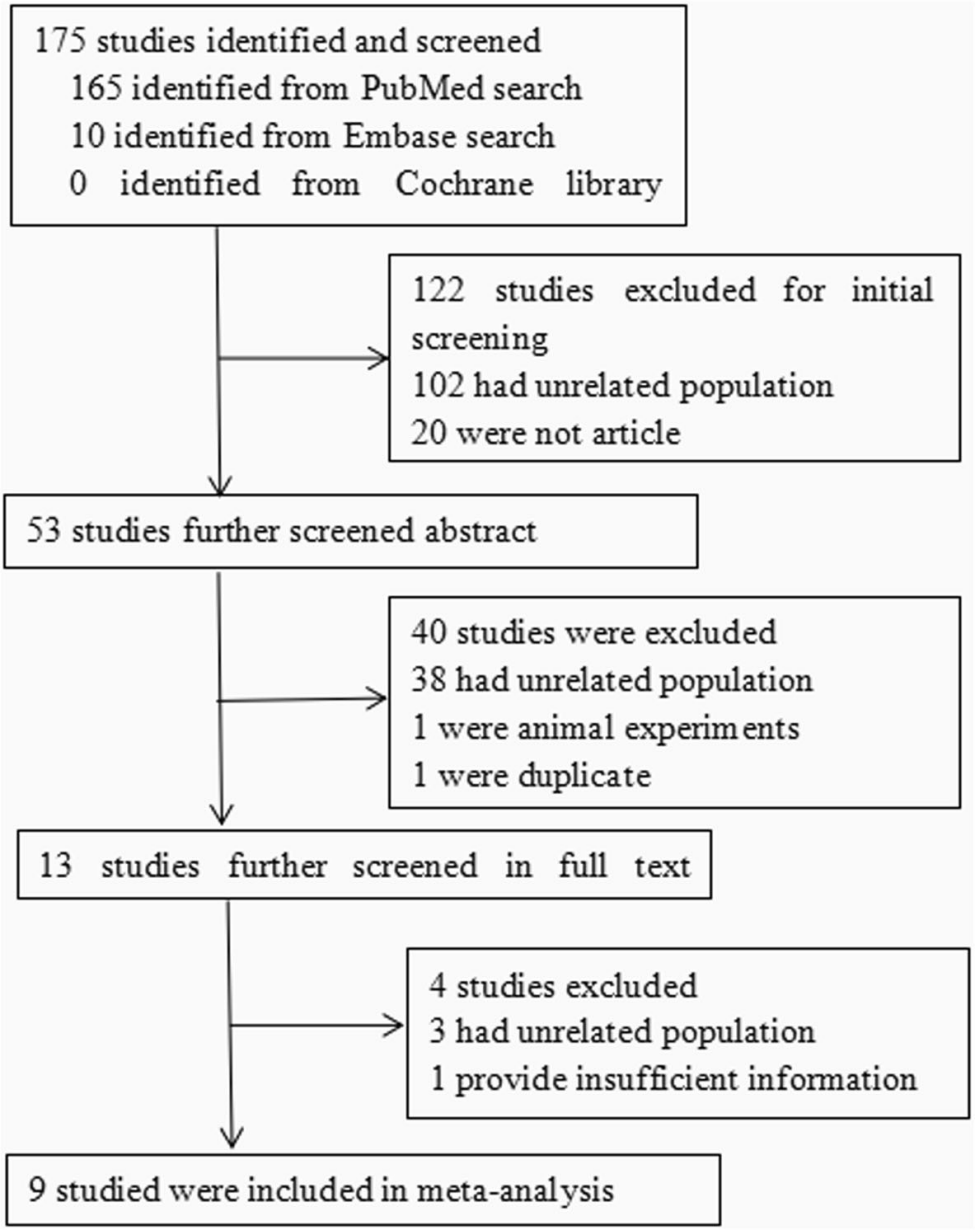
Flow chart of study selection

The inclusion criteria included randomized and nonrandomized studies reporting perventricular device closure of dcsVSDs. The exclusion criteria included case series already included in multicenter studies and case reports with sample sizes less than 10. Our search identified 175 articles, of which 166 were excluded (Fig. 1). A total of 9 articles [9–17] were included and further analyzed. Five studies were case series, and the other 4 studies were case-control studies, comparing perventricular device closure with surgical repair or transfemoral device closure.

This meta-analysis included 5 case series and 4 case-control studies. We used the Newcastle-Ottawa Scale (NOS) to assess the quality of the case-control studies. The NOS assesses the quality of studies based on the selection of the cases and controls (0–4 stars), the comparability of the cases and controls (0–2 stars) and the ascertainment of exposure (0–4 stars). NOS scores of > 6 stars are considered to indicate high quality [18]. We chose an 18-item, validated quality appraisal tool to evaluate the methodological quality of the case series. The quality assessments for each item were binary determinations of various aspects of the study, including the study objective, study population, intervention and cointervention, outcome measures, statistical analysis, results and conclusions, competing interests, and sources of support. High quality scores were ≥ 14 [19]. Disagreements in the quality assessment were resolved through discussion.

### Data extraction

Relevant data were extracted by two authors and entered into an electronic database. The data included publication details, including the publication year, first author name, weight, age, VSD size, sample size, device size, success number, complications number, length of intensive care unit (ICU) stay, length of hospital stay and median follow-up period. Successful device closure was defined as a residual shunt < 2 mm detected by TTE. Valvular regurgitation (including aortic/tricuspid/pulmonary regurgitation), residual shunting, and arrhythmias were considered permanent if they were reported and remained present at the time of the latest follow-up visit, regardless of severity. Residual shunting included all color jets observed across the VSD after deployment of the device. Data regarding other significant complications, such as death, device dislocation requiring reoperation, wound infection requiring reoperation, embolization, hemolysis, complete atrioventricular block (cAVB) and thromboembolism, were also extracted.

### Statistical analysis

Baseline characteristic data are presented as the median. Zero-event rates were approximated with [1/(4*sample size)] to allow calculation of the pooled occurrence rates. If a particular event was not reported in a study, then the study was excluded from the pooled analysis of these events [20].

We used a funnel plot of the sample size plotted against the operational success rate to evaluate the possibility of publication bias. The random-effects model was used to obtain the pooled estimates of the success rate and different types of complication rates. This study assumed that a total of 9 studies represented a random sample from the larger population of such studies. Each study had its own underlying effect size. The random-effects model assumed that there was a mean population effect size for which the study-specific effect varied. Thus, we could examine inter-study heterogeneity, such as differences in the study design type and definitions of success, as well as complications. We used the inconsistency statistic (I^2^) to evaluate the extent of heterogeneity. An I^2^ value greater than 50% was considered to indicate substantial heterogeneity. A 2-sided test at the 5% level was defined as indicating statistical significance, as determined using Stata version 15 (Stata Corp, College Station, TX, USA). Publication bias was tested using a funnel plot and Egger’s test. We further used a trim-and-fill method to estimate the number of missing trials if publication bias was evident.

## Results

### Publication bias

**Fig. 2:**
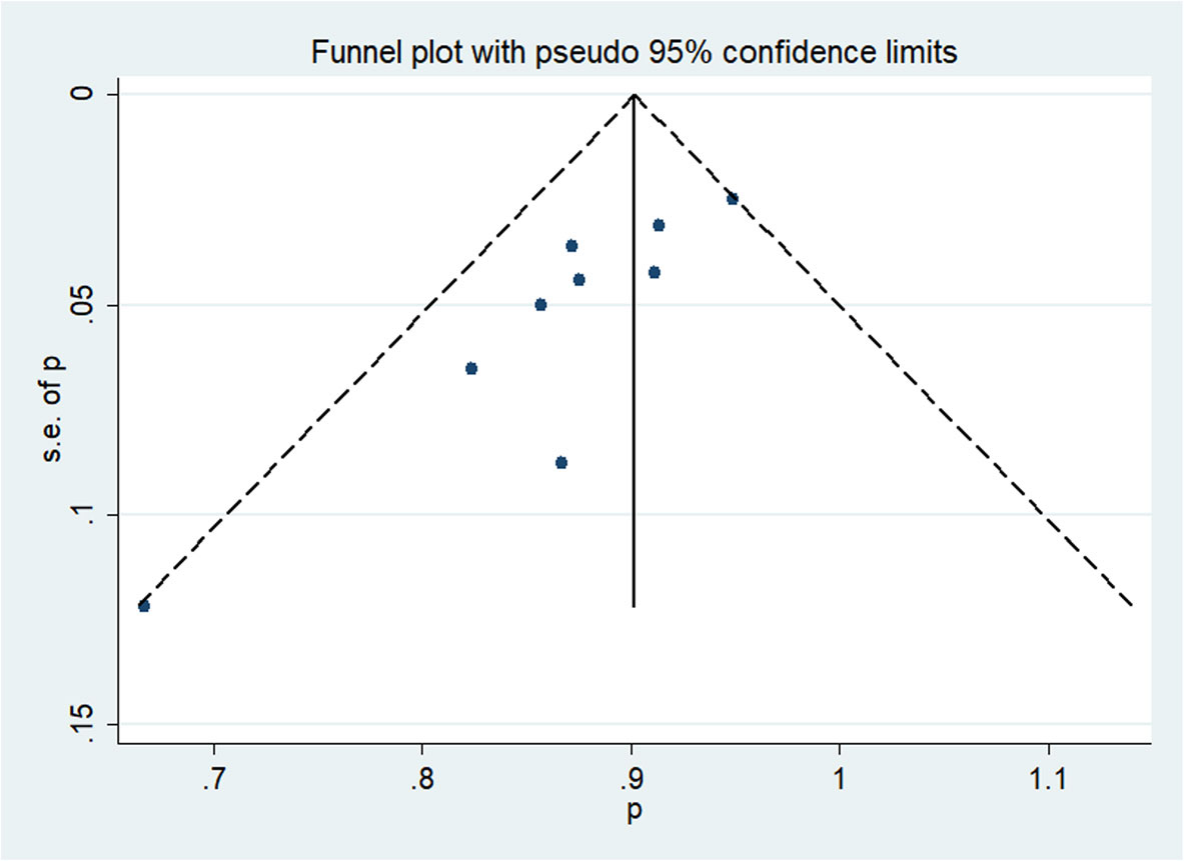
Funnel plot based on the operational success rate

**Fig. 3:**
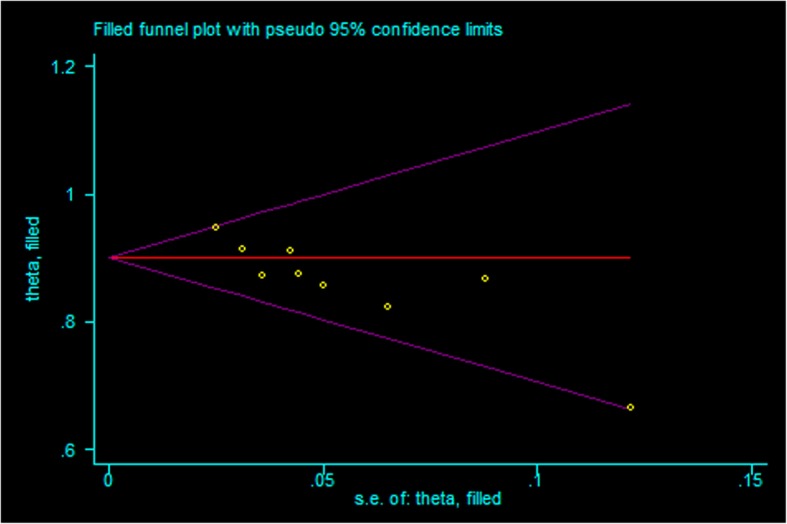
Funnel plot using the trim-and-fill method based on the operational success rate

A total of 9 studies (Table 1) investigated success and complication rates in 459 patients and were included in the analysis. The median follow-up duration ranged from 2 months to 5 years, with the mean age of patients ranging from 6.1 months to 4.5 years. The sex rate was reported in 5 studies, including 305 patients, 184 of whom were male. The pooled success rate was 0.89 (I^2^ = 26.5%, *P* = 0.208). Statistical evidence of publication bias was detected by a funnel plot (Fig. 2) and Egger’s and Begg’s test. The funnel plot showed funnel asymmetry, largely suggesting the presence of publication bias. The *P* value was 0.016 in Begg’s test, and *p* was 0.003 in Egger’s test, which suggested publication bias. We further used the trim-and-fill method to evaluate the publication bias. No trimming or filling was performed, and the 95% CI of the pooled operational success rate results was stable, which suggested that the publication bias was still acceptable (Fig. 3).

**Table 1:** Study characteristics

No.	First author	Published year	Study type	Quality	Sample size	Male	Age (years)	Weight (kg)	VSD size (mm)
1	Chen Qiang	2010	Case series	14	15	8	6.50	22.40	7.00
2	Ke Lin	2013	Case series	15	34	/	7.90	25.30	4.00
3	Lin Liu	2013	Case series	16	15	/	4.49	16.47	5.40
4	Shi Jun Hu	2014	case-control	7 stars	49	/	3.70	16.70	6.95
5	Da Zhu	2014	case-control	7 stars	56	/	4.36	16.80	5.03
6	Chen Zhao Yang	2015	case-control	7 stars	78	45	7.90	24.10	4.50
7	Shu Zhang	2015	Case series	15	86	54	7.00	22.50	4.80
8	Hua Cao	2016	Case series	14	81	38	10.50	22.50	6.40
9	Sijie Zhou	2017	case-control	8 stars	45	39	2.20	13.80	4.50

### Outcomes

**Fig. 4:**
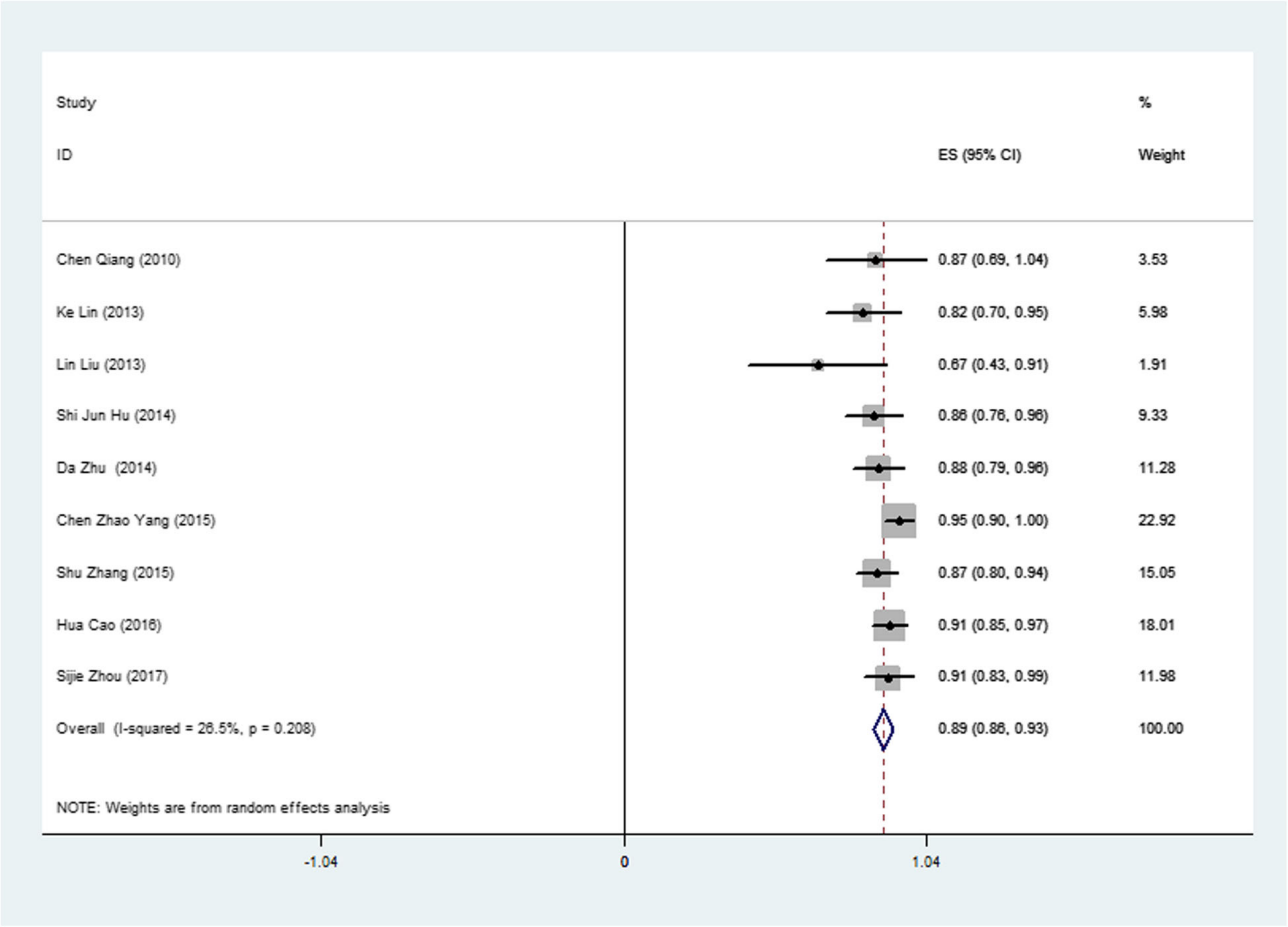
Forest plot of operational success rate

The success rate of perventricular device closure of dcsVSDs was moderate. Only 3 studies (sample size ranging from 45 to 81) reported a success rate of more than 90%. The Q statistic showed no evidence of substantial heterogeneity (I^2^ = 26.5%, *P* = 0.208), and we chose a fixed-effects model. The pooled estimate of the overall success rate of device closure in the 9 studies was 0.89 (95% CI: 0.86–0.93, I^2^ = 26.5%, *P* = 0.208) (Fig. 4). Further meta-regression analysis indicated no significant correlation between the success rate and the following factors: publication year, sample size, study type, mean age, mean weight, mean VSD size, ratio of device size/weight (all *P* > 0.05).

**Fig. 5:**
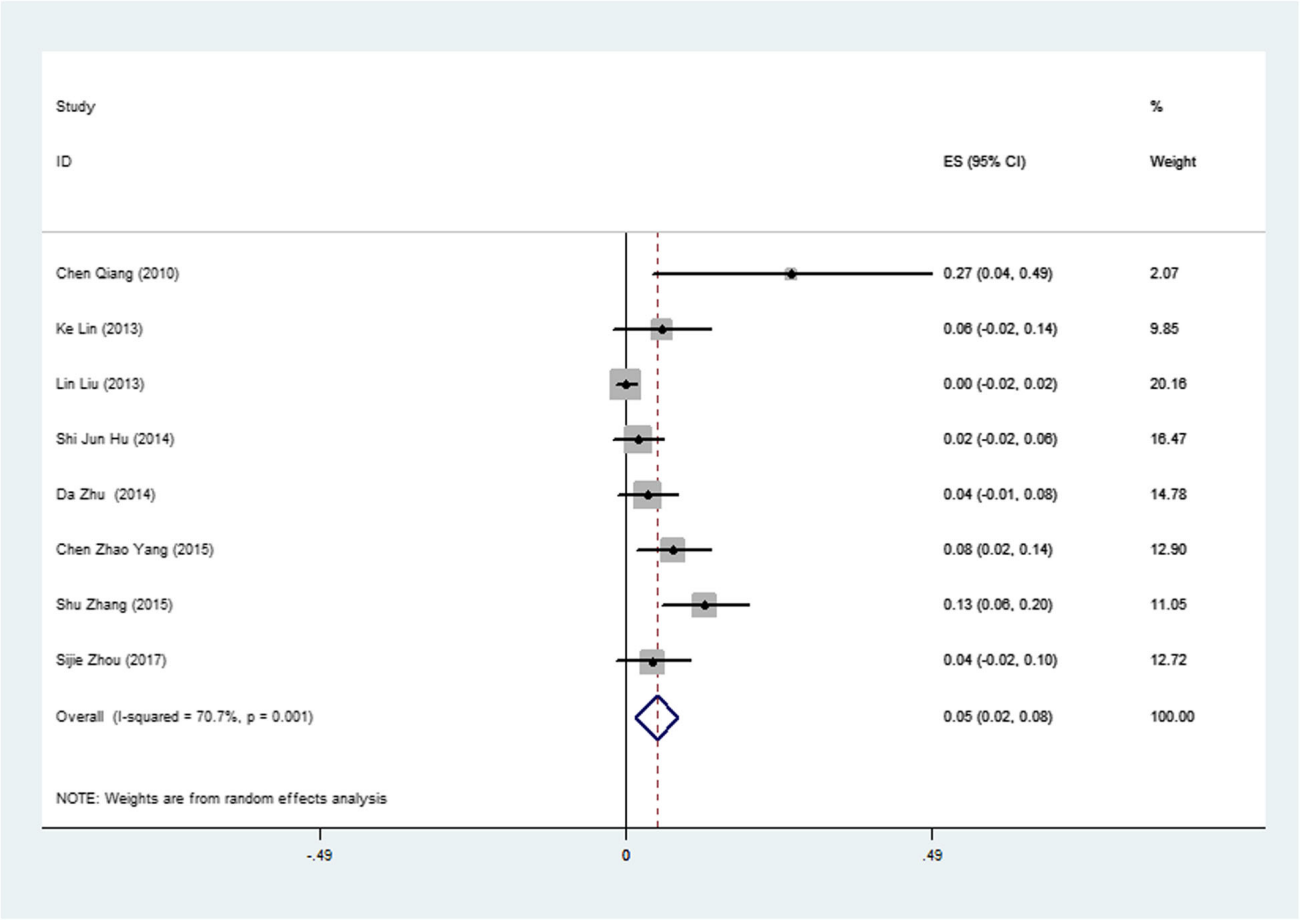
Forest plot of postoperative residual shunt rate

**Fig. 6:**
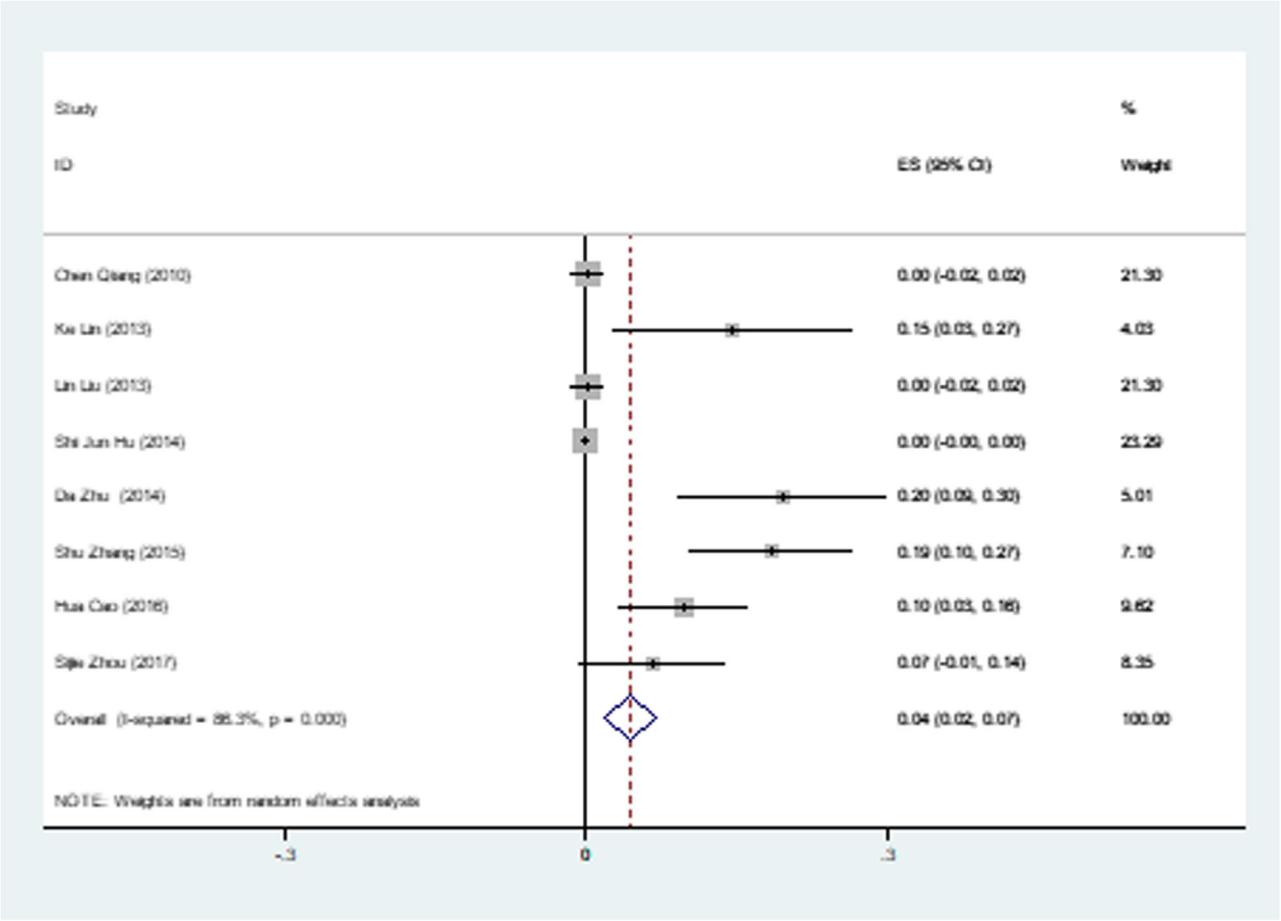
Forest plot of postoperative aortic regurgitation rate

**Fig. 7:**
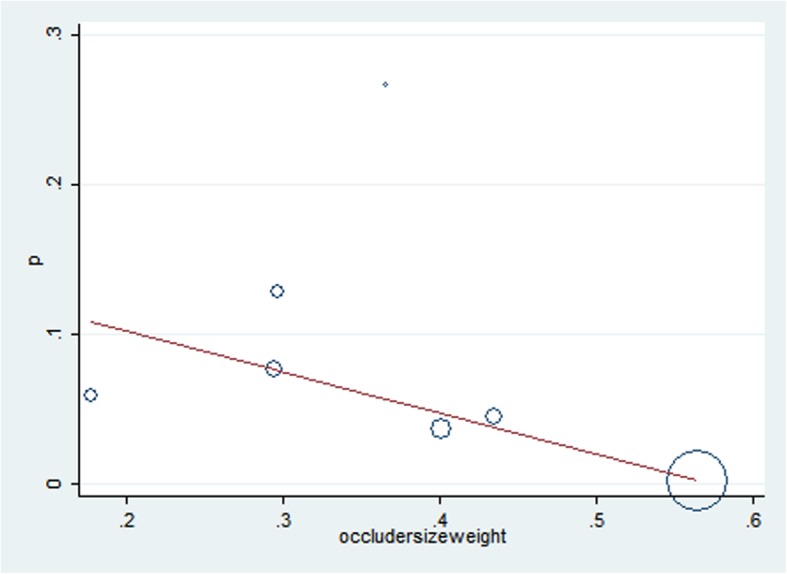
Scatter diagram of postoperative residual shunt

The most common minor complication was residual shunting, documented in 28 subjects among the 8 studies with 202 patients. The pooled rate of postoperative residual shunting was 0.05 (95% CI: 0.02–0.08, I^2^ = 70.7%, *P* = 0.001) (Fig. 5). The pooled rate of follow-up residual shunting was 0.000 (95% CI: − 0.001-0.001, I^2^ = 0.0%, *P* = 0.513), which meant almost all residual shunting disappeared during the follow up period. Another common minor complication was trivial to mild aortic regurgitation (AR), documented in 43 subjects among the 8 studies with 381 patients. The pooled rate of postoperative AR was 0.045 (95% CI: 0.018–0.071, I^2^ = 50.96%, *P* = 0.000) (Fig. 6). The pooled rate of follow-up AR was 0.001 (95% CI: − 0.003-0.004, I^2^ = 63.00%, *P* = 0.009). The pooled rates of postoperative and follow-up minor complications are shown in Table 2. Further regression showed that a ratio of occluder size (mm)/weight (kg) above 0.4 was a risk factor for a postoperative residual shunt, with a Coef of − 0.28 (95% CI: − 0.49- -0.66, *P* = 0.020) (Fig. 7), and follow-up pulmonary regurgitation, with a Coef of − 0.83 (95% CI: − 0.15- -0.16, *P* = 0.026), which suggested that the ratio of occluder size (mm)/weight (kg) below 0.4 may be another patient selection criterion for perventricular device closure of a dcsVSD.

**Table 2:** The pooled rate of minor postoperative and follow-up complications

Pooled events	Events(n)	%	Included studies	Incidence(95%CI)	Heterogeneity(I^2^)	*p*
Post-operative minor complications	106	100.00	9	0.208 (0.119–0.297)	95.00	0.000
Trivial to mild aortic regurgitation	43	40.57	8	0.045 (0.018–0.071)	50.96	0.000
Trivial to mild residual shunt	28	26.42	8	0.050 (0.015–0.085)	74.50	0.000
Trivial to mild tricuspid regurgitation	22	20.75	7	0.002(−0.004–0.008)	77.30	0.000
Trivial to mild pulmonary regurgitation	13	12.26	7	0.002(− 0.004–0.008)	57.10	0.030
Follow-up minor complications	44	100.00	9	0.004(−0.003–0.011)	85.90	0.000
Trivial to mild aortic regurgitation	17	38.64	8	0.001(−0.003–0.004)	63.00	0.009
Trivial to mild pulmonary regurgitation	15	34.09	7	0.003(−0.004–0.009)	63.30	0.012
Trivial to mild residual shunt	7	15.91	9	0.000(−0.001–0.001)	0.00	0.513
Trivial to mild tricuspid regurgitation	5	11.36	7	0.000(−0.001–0.001)	0.00	0.479

A total of 53 patients were converted to conventional surgical repair in 8 studies. The reasons for conversion to surgical repair under CPB included significant mild to significant AR (47.83%), a significant residual shunt (RS) (28.26%), device dislocation (17.39%) and failure to establish a path (6.52%). No severe intraoperative severe arrhythmias, including complete atrioventricular blocks (cAVBs), were reported in the enrolled studies, either postoperatively or in the follow-up period. The pooled rates of severe intraoperative, postoperative and follow-up complications are shown in Table 3.

**Table 3:** The pooled rate of severe intra-operative, postoperative and follow-up complications

Pooled events	Events(n)	%	Included studies	Incidence(95%CI)	Heterogeneity(I^2^)	*p*
Intra-operative severe complications	53	100	9	0.106 (0.073–0.140)	70.7	0.208
newly aortic regurgitation	22	47.83	8	0.049 (0.014–0.84)	70.5	0,001
significant residual shunt	13	28.26	8	0.016 (0.003–0.029)	16.3	0.302
device mal-position	8	17.39	8	0.000(−0.001–0.001)	16.4	0.301
failure in establishing track	3	6.52	8	0.000(−0.001–0.001)	0.0	0.871
severe arrhythmias	0	0.00	9	0.000 (0.000–0.000)	0.0	1.000
Post-operative severe complications	4	100.00	9	0.000(−0.001–0.001)	0.0	0.847
Occluder dislocation	2	50.00	9	0.000(−0.001–0.001)	0.0	0.979
Left ventricular outflow tract obstruction	1	25.00	9	0.000(−0.001–0.001)	0.0	0.998
wound infection requiring reoperation	1	25.00	9	0.000(−0.001–0.001)	0.0	0.998
Mild to significant aortic reguriation	0	0.00	9	0.000 (0.000–0.000)	0.0	1.000
Mild to significant tricuspid regurgitation	0	0.00	9	0.000 (0.000–0.000)	0.0	1.000
Severe arrhythmias	0	0.00	9	0.000 (0.000–0.000)	0.0	1.000
Follow-up severe complications	0	0.00	9	0.000 (0.000–0.000)	0.0	1.000
Severe arrhythmias	0	0.00	9	0.000 (0.000–0.000)	0.0	1.000
Mild to significant aortic reguriation	0	0.00	9	0.000 (0.000–0.000)	0.0	1.000
Mild to significant tricuspid regurgitation	0	0.00	9	0.000 (0.000–0.000)	0.0	1.000
Mild to significant residual shunt	0	0.00	9	0.000 (0.000–0.000)	0.0	1.000

## Discussion

Perventricular device closure of perimembranous VSDs (pmVSDs) and muscular VSDs (mVSDs) has been confirmed to be safe and effective [20, 21]. Recently, this technology has also been used in patients with dcsVSDs. However, due to the special location of dcsVSDs, the safety and efficacy of perventricular device closure of dcsVSDs is still unclear. In this systematic review, we have attempted to evaluate the efficacy and safety of this technology.

The included studies were 5 case series and 4 case-control studies of high quality. The invasive procedure limited the blinding of the participants and resulted in a lack of RCTs. We attributed the funnel asymmetry to publication bias. Studies with promising results had an increased likelihood of being accepted and published. Thus, publication bias may contribute to a higher pooled success rate. Fortunately, the trim-and-fill test did not show any trimming or filling, and the results were stable, which suggested that the bias was acceptable.

We defined operational success as patients without fatal or severe early-term or late-term complications. The pooled success rate was 0.89 (95% CI: 0.86–0.93, I^2^ = 26.5%, *P* = 0.208) for 9 studies with 459 patients. Further meta-regression analysis indicated no significant correlation between the success rate and the following factors: publication year, sample size, study type, mean age, mean weight, mean VSD size, and ratio of device size/weight, which may indicate the short learning curve and promotability of this technology. Compared with surgical repair, perventricular device closure does not require CPB. Compared with the transfemoral approach, the perventricular approach provides direct access and facilitates manipulation of the device position and orientation during device deployment. We attributed this to the shorter delivery path. A shorter delivery path also minimizes the risk of intracardiac structural damage due to catheter friction or rubbing. Thus, for experienced cardiac surgeons, the learning curve is short, and the promising prospects of this technology are easily promoted.

Only patients with isolated dcsVSDs were included, and patients with other coexisting cardiac anomalies requiring surgical intervention, severe pulmonary hypertension, or significant aortic prolapse and VSDs larger than 10 mm were excluded. However, no uniform patient inclusion criteria were applied among the cardiac centers. Whether patients with VSD sizes between 5 mm and 10 mm or patients with mild aortic valve prolapse (AVP) could undergo this procedure is unclear. Most studies recommended that the VSD size should be less than 10 mm. However, Cao and his colleagues recommended that the VSD size should be less than 5 mm. They found that in cases with a VSD size > 5 mm, the effective contact area of the occluder became small and, in cases with a VSD size < 5 mm, there was a relatively small opening in the superior margin. Thus, in cases with a VSD size < 5 mm, the device rarely affects the pulmonary valve and is less likely to be displaced from the original position [16]. The incidence of occluder displacement in patients with VSDs > 5 mm was greater than the incidence of displacement in patients with VSDs < 5 mm. Hu and his colleagues also reported that a dcsVSD size ≥5 mm was a predictor of percardiac device closure failure (odds ratio, 41.25; 95% confidence interval, 4.69–362.72; *P* < 0.001) [12]. Meta-regression analysis showed no correlation between the success rate and mean VSD size. Patients with VSD sizes between 5 mm and 10 mm may still be suitable for this procedure. Most studies did not exclude patients with AVP (below severe). However, multivariate logistic regression performed by Zhang and his colleagues showed that procedure failure was associated with the occurrence of preoperative AVP (even in mild degree). The AVP may lead to underestimation of the VSD size and increase the risk of device dislocation. The interface between the aortic valve and device may cause procedure failure as well as procedure-induced valve complications. Unfortunately, most enrolled patients did not provide enough information to conduct further analyses [15].

The pooled rate of severe intraoperative complications was 0.106 (95% CI: 0.073–0.140, I^2^ = 70.7%, *P* = 0.208). A total of 53 patients were converted to conventional surgical repair, including 22 patients with new mild to significant aortic regurgitation, 13 patients with significant residual shunting, 8 patients with device dislocation, and 3 patients with failure to establish a path. New mild to significant aortic regurgitation and significant residual shunting were the most common reasons for conversion. The pooled rates of failure to establish a path, device dislocation and severe arrhythmias were low in perventricular device closure of dcsVSDs. The reason for the low incidence of failure to establish a path was that such a procedure could provide a perpendicular approach via the right ventricular surface toward the dcsVSD. Suitable puncture was determined by depressing the right ventricular free wall with an index finger to find the strongest tremor site under transesophageal echocardiography (TEE) guidance. Most complications disappeared after removal of the device, which suggested the importance of choosing a suitable device size. The asymmetrical occluder was the most widely used occluder in these studies. The device size was selected according to the TEE measurement and allowed a margin of 0 to 2 mm in excess of the diameter of the VSD. Suitable device size is a key factor in preventing procedure-related AR. Lin and his colleagues reported that the ratio of the device diameter to weight and procedure-related AR had a statistical association (OR = 4158.325, 95% CI 4.388–3,941,113.209, *P* = 0.017) [10]. Although the pooled rate of device dislocation was only 0.000 (95% CI: − 0.001-0.001, I2 = 16.4%, *P* = 0.301), TEE was necessary to assess the presence of the device position for 10–20 min after device placement [18].

The pooled rate of severe postoperative complications was promising, only 0.000 (95% CI: − 0.0010-0.001, I2 = 0.0%, *P* = 0.847). A total of 2 patients required re-operation, including one for device dislocation and the other for wound infection. Device dislocation may be a procedure-related complication caused by a lack of experience. No cases of new mild or significant aortic regurgitation were observed. The pooled rates of mild-to-significant aortic/tricuspid regurgitation and severe arrhythmias were 0.000 (95% CI: 0.000–0.000, I2 = 0.00%, *P* = 1.0). The pooled rate of severe complications in the follow-up period was 0.000 (95% CI: − 0.000-0.000, I2 = 0.0%, *P* = 1.00). cAVB is a severe complication during and after device closure for a VSD, especially in cases with perimembranous VSD (pmVSD) [19, 20]. However, no cAVB occurred in the enrolled studies, and the pooled rate was 0.00 (0.00–0.00) (95% CI: − 0.000-0.000, I2 = 0.0%, *P* = 1.0). We attributed this to the relatively long distance between the conduction tissue and the rim of the dcsVSD. The posteroinferior margin of the dcsVSD is usually well separated from the tricuspid valve annulus by a band of muscle [22]. Thus, the chance of conduction system injury from mechanical trauma compression by the delivery system or device seems to be small [23]. However, we should still pay attention to late cAVBs resulting from chronic inflammation or fibrosis.

Minor complications include trivial to mild residual shunts (RSs), AR, pulmonary regurgitation (PR). Most studies couldn’t provide information regarding intraoperative minor complications. Thus, we would discuss the minor postoperative complications and its outcome in follow-up period.

The pooled rate of postoperative residual shunts was 0.050 (95% CI: 0.050–0.085; I^2^ = 74.5%, *P* = 0.000). However, most of them disappeared during the follow-up period, and the pooled rate of follow-up RSs was 0.00 (95% CI: − 0.001-0.001; I^2^ = 0.00%, *P* = 0.51). This change means that most RSs could close spontaneously during the follow-up period. We attribute this to endothelialization covering the surface of the device and neointima forming several weeks after operation [24]. Further meta-regression showed that a ratio of occluder size (mm)/weight (kg) above 0.4 was a risk factor for postoperative RSs with a Coef of − 0.28 (95% CI: − 0.49- -0.66, *p* = 0.020). Zhang and his colleagues also reported that procedure-induced aortic regurgitation (AR) was associated with device diameter and patient weight (OR = 12.3 95% CI 1.5–99.2) [16]. Thus, the ratio of occluder size (mm)/weight (kg) below may be another patient selection criterion for perventricular device closure of dcsVSDs.

The pooled rate of postoperative AR was 0.045 (95% CI: 0.018–0.071, I^2^ = 50.96%, *P* = 0.00). Further meta-regression showed that a ratio of VSD size (mm)/weight (kg) above 0.3 was a risk factor for postoperative AR, with a Coef of − 0.14 (95% CI: − 0.20-0.87, *P* = 0.001), which suggested that patients with a low body weight and a relatively large VSD size are more likely to experience postoperative AR. Most of the AR disappeared during the follow-up period, and the pooled follow-up rate of AR was 0.001 (95% CI: − 0.003-0.004, I^2^ = 63%, *P* = 0.009). However, new AR also occurred during the follow-up period. The position of the aortic cusp changes throughout the cardiac cycle. During the diastolic phase, the mid portion of the cusps will be pushed below the annulus level by the diastolic blood pressure. Although the device has a 0-mm superior rim, the interface between the device and the aortic cusps may be unavoidable after endothelialization during the follow-up period.

The pooled rate of trivial to mild postoperative pulmonary regurgitation (PR) was 0.002 (95% CI: − 0.004-0.008, I^2^ = 57.10%, *p* = 0.030). However, the pooled rate of trivial to mild pulmonary regurgitation was 0.003 (95% CI: − 0.004-0.009, I^2^ = 63.30%, *p* = 0.012). The pooled rates of trivial to mild postoperative/follow-up pulmonary regurgitation were similar. Although the pulmonary system has a relatively low pressure, trivial to mild PR is acceptable. However, a longer follow-up may be necessary to assess the true impact on pulmonary valve function. Further student t-regression showed that a ratio of occluder size (mm)/weight (kg) above 0.4 was a risk factor for follow-up PR with a Coef of − 0.28 (95% CI: − 0.49- -0.66, *P* = 0.020), which suggested that a ratio of occluder size (mm)/weight (kg) below 0.4 may be another patient selection criterion for perventricular device closure for dcsVSDs.

### Study limitations

First, there was a publication bias in this study, though it was acceptable. Second, the number of enrolled patients was limited, and the follow-up period in the enrolled studies was variable. Third, several studies enrolled in the meta-analysis did not provide sufficient information. Most studies did not provide preoperative data regarding the subarterial rim, the degree of aortic regurgitation, or the degree of aortic prolapse. Thus, it is difficult to analyze whether the above factors affect the success rate or complication rate. The indications and contraindications for this procedure remain unclear. Some studies reported all cases of valvular regurgitation in detail, while others only reported cases of severe valvular regurgitation. The follow-up period was different in each study. Thus, it is difficult to define transient or permanent; we only enrolled cases reported at the final follow-up review as being permanent, and we recorded all other cases as being transient. Third, this analysis only included case series and case-control studies but no randomized controlled studies. To achieve a consensus on indications, further studies should include a larger number of cases with longer follow-up periods and sufficient data to determine the risk factors for procedure failure.

## Conclusion

Perventricular device closure may be an alternative to conventional surgical repair in selected patients with dcsVSDs. The success rate wasn’t related to publication time and sample size, suggesting a relatively short learning curve and the technique’s potential for application. The incidence of severe complications during the hospital stay and follow-up was low, especially for severe valvular regurgitation, device dislocation and cAVB. However, the indications and contraindications are still doubtful. Patients with a VSD size of 5 mm^− 10^ m may be suitable for this procedure. Additionally, a ratio of occluder size (mm)/weight (kg) above 0.4 may be another contraindication. Further studies should include a larger number of cases with longer follow-up periods and sufficient data to determine the risk factors for procedure failure.

## Data Availability

Data sharing not applicable to this article as no data sets were generated or analyzed during the current study.

## Abbreviations

AR: Aortic regurgitation
AVP: Aortic valve prolapse
cAVB: Complete atrioventricular block
CPB: Cardiopulmonary bypass
dcsVSD: Doubly committed subarterial ventricular septal defects
ICU: Intensive care unit
NOS: Newcastle-Ottawa Scale
pmVSD: Perimembranous VSD
RS: Residual shunt
TEE: Transesophageal echocardiography
TR: Tricuspid regurgitation
TTDC: Transthoracic device closure
TTE: Transthoracic echocardiography
